# Cannabidiol Cigarettes as Adjunctive Treatment for Psychotic Disorders – A Randomized, Open-Label Pilot-Study

**DOI:** 10.3389/fpsyt.2021.736822

**Published:** 2021-11-04

**Authors:** Patrick Köck, Elisabeth Lang, Valerie-Noelle Trulley, Frieder Dechent, Katja Mercer-Chalmers-Bender, Priska Frei, Christian Huber, Stefan Borgwardt

**Affiliations:** ^1^University Psychiatric Clinics Basel, University of Basel, Basel, Switzerland; ^2^Department of Psychiatry and Psychotherapy, University of Lübeck, Lübeck, Germany; ^3^Department of Biomedical Engineering, Institute of Forensic Medicine, University of Basel, Basel, Switzerland

**Keywords:** cannabis, schizophrenia, substance-related disorders, comorbidity, antipsychotic agents

## Abstract

**Background:** Psychotic disorders are associated with high rates of comorbid substance use disorders. Use of cannabis rich in tetrahydrocannabinol (THC) is linked to an increased risk of psychosis, worsening of psychotic symptoms, and an adverse course of psychotic disorders. Previous studies suggest oral cannabidiol (CBD) as possible novel antipsychotic agent; however, no studies evaluated the effects of smoked CBD.

**Objective:** The main aim of the study was to clarify the antipsychotic potential of CBD used as adjunctive therapy simulating a naturalistic setting. Our trial is the first study evaluating the effects of smoked CBD-cigarettes as adjunctive therapy for psychotic symptoms.

**Methods:** A randomized, placebo-controlled open-label trial of cigarettes containing CBD-rich cannabis (THC < 1%) as adjunctive therapy to standard psychiatric treatment was conducted (ClinicalTrials.gov identifier NCT04700930). Primary outcomes were mean scores of Positive and Negative Syndrome Scale (PANSS), Brøset Violence Checklist, the Beck's Depression Inventory (BDI), the Subjective Well-Being Under Neuroleptics Scale short form (SWN-K), and antipsychotic medication equivalent doses. Outcomes were assessed after 4 weeks of acute treatment and long-term follow-up after discontinuation of CBD-cigarettes after 25 weeks. Participants were 31 acutely psychotic patients with tobacco use disorder and a mean age of 35.1 ± 10.58 years (71% male). Comorbid cannabis use was diagnosed in 51.6%.

**Results:** A discontinuous multilevel model revealed no significant group differences for primary outcomes. After 4 weeks of acute treatment, mean PANSS and BDI decreased in both groups, while an increase of antipsychotic medication equivalent was observed in the placebo group.

**Conclusions:** The presented findings might suggest an antipsychotic medication sparing effect of CBD-cigarettes as adjunctive treatment of acute psychosis. However, the low number of participants did not allow for further statistical analysis. Hence, a larger study sample and a more rigorous study design (blinding of the interventional product, fixed dosing regimen) may reveal different results.

**Clinical Trial Registration:** ClinicalTrials.gov, identifier: NCT04700930

## Introduction

The gold standard for the treatment of schizophrenia consists of antipsychotic drugs. Those mainly act antagonistically upon the dopamine receptors (1). However, short-term non-response and non-remission to routine pharmacotherapy are frequent. About 40% of patients discontinue antipsychotic treatment within a year and up to over 70% within 18 months (2). A Finnish 20-year follow-up study showed that long-term antipsychotic treatment is associated with increased survival (3). Moreover, treatment success seems significantly diminished with comorbid SUDs (4, 5). Generally, psychiatric disorders are associated with higher substance use disorder (SUD) rates (6). There is sound evidence for a high prevalence of the comorbidity between schizophrenia-spectrum disorders or other psychiatric conditions and SUDs (7–9). Robust physiological and epidemiological evidence supports the link between schizophrenia and cannabis use (10).

While tetrahydrocannabinol (THC) can cause temporary and dose-dependent psychotic symptoms (11), interestingly, some clinical findings suggest differential effects of another cannabinoid, cannabidiol (CBD). A favorable impact of CBD has been demonstrated in patients with schizophrenia, psychotic symptoms, or people who are at high risk of psychosis (12–15). Leweke et al. (15) performed a double-blind, randomized clinical trial (RCT) to assess the effects of amisulpride vs. CBD (800 mg amisulpride, *N* = 19; vs. 600 mg CBD, *N* = 20). Both medications showed similar, significant antipsychotic efficacy (reduction of PANSS), but CBD displayed a superior side effect profile (15). In McGuire et al. (12) reported their findings of a double-blind RCT. Participants (CBD group *N* = 43, placebo group *N* = 45) received either CBD 1,000 mg/d or placebo as an adjunction to their pre-established, regular antipsychotic medication. The study found lower levels of positive psychotic symptoms in the CBD-group. Boggs et al. (16) studied the effects of CBD augmentation (600 mg/day, p.o.) in stable participants (*N* = 36) with neuroleptic medication for chronic schizophrenia. PANSS scores did not improve over 6 weeks compared to placebo (12). Bhattacharyya et al. (14) researched the effects of a single oral dose of CBD (600 mg, p.o.) in medication-naive participants at clinical high risk of psychosis (*N* = 16 CBD, *N* = 17 placebo, *N* = 19 healthy controls). Additionally, fMRI data might indicate a partial normalization in striatal, parahippocampal, and midbrain function compared to the placebo group (14). As encouraging as previous study outcomes have seemed, recent systematic reviews found only mixed evidence supporting the antipsychotic efficacy of CBD and call for further, more extensive investigations (17). Despite the debatable effectiveness of CBD in psychotic disorders, treatments with CBD displayed a favorable side effect profile relative to standard pharmacological therapies (18, 19). Another systematic review suggests CBD treatment as a promising intervention for psychotic disorders and comorbid SUDs. Thus, CBD implementation might offer an innovative harm-reduction approach and add-on therapeutic strategy for comorbid patients, but clinical studies are needed (4). Trial duration with oral CBD to treat neuropsychiatric disorders ranged between 4 to 6 weeks, and doses ranged from 40 mg to 1,000 mg/day (17). A consensus regarding study design is still absent. The present open-label study aimed at evaluating the antipsychotic effects of smoked CBD as adjunctive therapy to standard psychiatric treatment.

After THC, CBD is the best-studied phytocannabinoid (20). CBD interacts with multiple receptor systems, but the exact mechanisms of its suggested effects are poorly understood. Unlike THC, CBD is an inverse agonist on the CB2-cannabinoid receptor and a non-competitive modulator on the CB1-cannabinoid receptor. In contrast to most antipsychotic medications, CBD does not seem to possess dopamine receptor antagonistic qualities (21–23). CBD activates the 5HT_1A_ receptor, inhibits adenosine reuptake, and increases the endocannabinoid anandamide (24). The inhibition of glutamate release and partial agonism of the DA_2_ dopamine receptor also seem essential, considering the proclaimed antipsychotic effects (25).

In Switzerland, cannabis flowers are only classified as narcotics if they contain 1% or more THC. As a result, CBD-rich cannabis flowers with THC concentrations below 1% are available in Swiss tobacco shops or supermarkets. The intervention product used in this study is commercially available in Switzerland. The promising effects of CBD in schizophrenia by previous clinical studies (12, 15) inspired the following research design. Moreover, the concept of harm-reduction seemed rational concerning the highly prevalent cannabis use in psychotic patients. The presented study hypothesized that cigarettes containing CBD-rich cannabis (<1% THC), as adjunctive therapy in standard psychiatric treatment of acute psychosis, would reduce psychotic symptoms, depressive symptoms, and violent behavior. Secondly, it was hypothesized that such CBD-cigarettes would increase subjective well-being and decrease necessary antipsychotic medication. In the following, the results of an open-label pilot study investigating the effects of cigarettes containing CBD-rich cannabis as adjunctive intervention in treating psychosis and comorbid tobacco use disorder are described. A high drop-out rate and the open-label study design compromise reliability of the results. For terms of better readability, we refer to cigarettes containing CBD-rich cannabis as “CBD cigarettes” throughout the text.

## Methods

### Study Design

**Figure 1:**
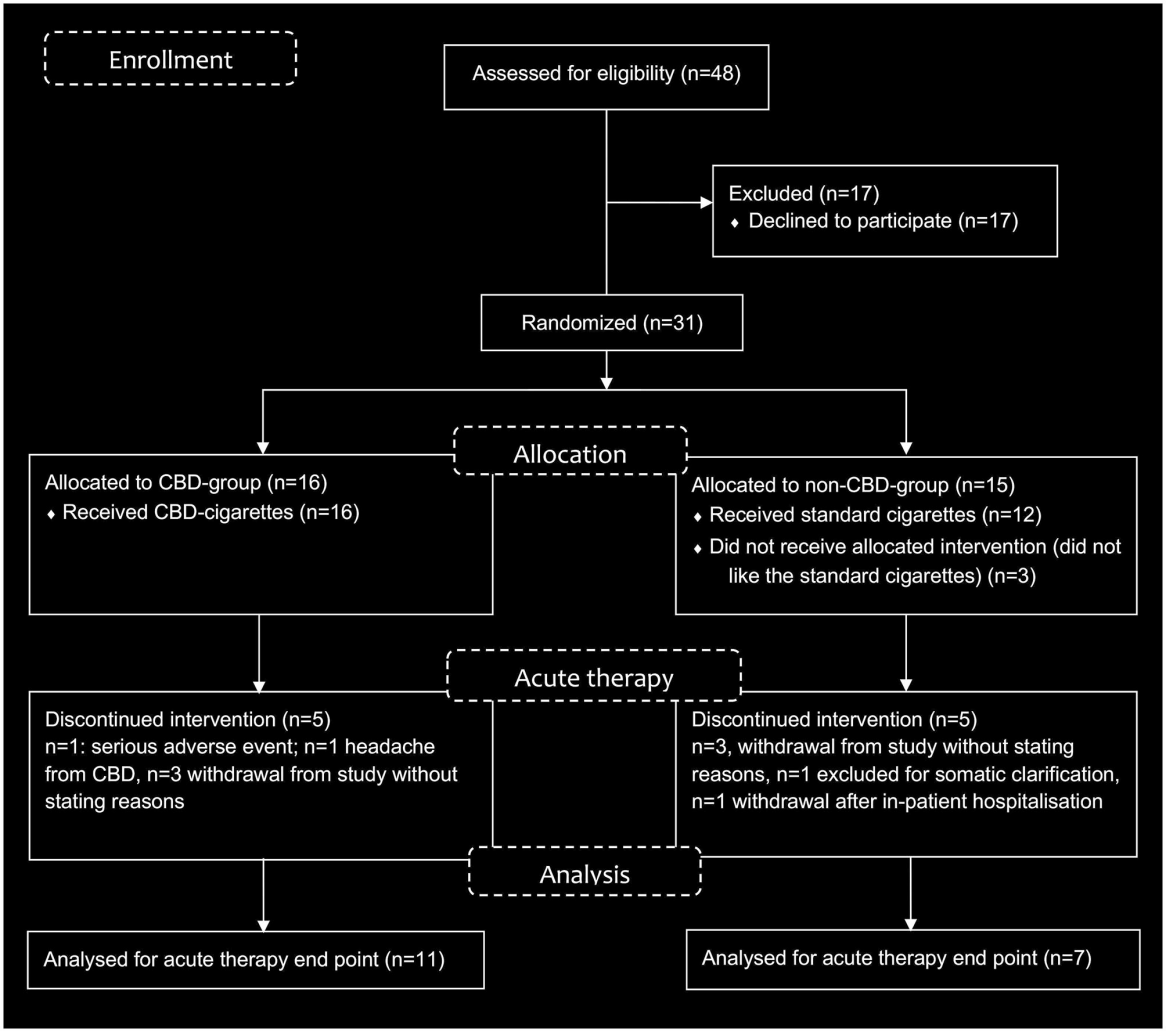
Study flow diagram.

An open-label, randomized, placebo-controlled study of CBD-cigarettes as adjunctive therapy in 31 acutely psychotic patients with schizophrenia or psychotic disorders and comorbid tobacco use disorder was conducted. The study intervention consisted of handing out CBD-cigarettes to the *verum*-group (CBD-group) and standard tobacco cigarettes to the *placebo*-group (non-CBD-group), in addition to routine psychiatric treatment (see Figure 1). Participants received either CBD- or standard cigarettes as on-demand medication during the acute therapy phase (day 0–28). There were mainly two reasons for this decision. Firstly, instructing the participants to smoke a certain fixed number of cigarettes deemed impossible. The day-to-day amount of CPD varies on average only about 2.5 cigarettes in the general smoking population [Hughes et al. (26)]. Contrary, considering our clinical experience and limited research data, admission to a psychiatric hospital and the treatment of acute mental illness typically interfere with pre-hospitalization smoking habits (27). Ker and Owens (27) found an overall increase in tobacco consumption from 5 to 13 CPD in psychiatric inpatients (27). Secondly, our study investigated the effects of an open-market product. Thus, we provided CBD-cigarettes on-demand to simulate naturalistic conditions within acute inpatient psychiatric treatment.

Our study group examined patients on days 0, 7, 14, 21, and 28 while acutely hospitalized (acute therapy phase, days 0–28). We performed follow-up assessments on day 91 and day 175. Hence, the study consisted of 7 visitations (V1–V7) within a 25-week duration. Included were men and non-pregnant women, age 18–65 years, with affective or non-affective psychotic disorders. Excluded were non-smokers, pregnant or breastfeeding women, and patients with organic psychotic diseases. All participants signed written informed consent. The Swiss Ethics Committee (Ethikkommission Nordwest- und Zentralschweiz, EKNZ) approved the study, and permission was filed under Project ID 2018-01111. Consecutively the study was registered at ClinicalTrials.gov by the identifier NCT04700930. Our study group carried out the first assessment and patient inclusion on October 4, 2018. On January 6, 2020, we completed the last subject assessment.

### Interventional Product

CBD-cigarettes were obtained by the swiss tobacco manufacturer “Heimat” (Koch and Gsell AG, 9323 Steinach, Switzerland). According to the manufacturers' statement, one package of 20 CBD-cigarettes contained 4 grams of swiss hemp, with a concentration of 10% CBD and <1% THC. The cigarettes consisted of 20% hemp and 80% tobacco. Hence, a single CBD-cigarette contained ~ 20 mg of CBD. We obtained the standard cigarettes with only tobacco (*placebo*) from the “Heimat” company. Due to the intensive odor of the CBD-cigarettes, we opted against blinding procedures. Therefore, participants and study staff would easily distinguish the CBD-cigarettes from the standard cigarettes when handling the intervention products.

### Participants

Participants were 31 acutely psychotic patients (22 males; nine females) that entered our psychiatric clinic as inpatients and met pre-established eligibility criteria. In our study sample the mean PANSS baseline score was 79.30 (SD = 16.97), indicating moderate mental impairment according to Leucht et al. (28). All study subjects were tobacco smokers, while the average amount of smoked cigarettes was 22.3 cigarettes per day (SD = 8.33). The self-reported mean cannabis use was 1.97 (SD = 3.1) joints per day. The average age at study initiation was 35.1 (SD = 10.58) years. In the study sample, 23 had schizophrenia, four were diagnosed with schizoaffective disorder, one individual had an acute polymorphic psychotic disorder with symptoms of schizophrenia, two had a bipolar disorder with psychotic symptoms, and one was diagnosed with a psychotic disorder due to cannabis use. Comorbid Cannabis Use Disorder was diagnosed in 51.6% of the study sample (*N* = 16). 12.9% had comorbid Alcohol Use Disorders (*N* = 4), 9.7% had Cocaine or Stimulant Use Disorders (*N* = 3), and in one subject, a comorbid Hallucinogen Use Disorder (*N* = 1) was diagnosed. All participants were randomized, either into the CBD-group (*verum*) or the standard cigarette-group (non-CBD, *placebo*). For detailed participant information, see Tables 1, 2.

**Table 1:** Overview of participant data.

	**Non-CBD**		**CBD**		**Total**	
	**(*****N*** **=** **15)**		**(*****N*** **=** **16)**		**(*****N*** **=** **31)**	
***Psychotic disorders***	***n***	***%***	***n***	***%***	***n***	***%***
Schizophrenia	11	35%	12	39%	23	74%
Schizoaffective disorder	1	3%	3	10%	4	13%
Polymorphic psychotic disorder	1	3%	0	0%	1	3%
Bipolar disorder/psychotic symptoms	2	6%	0	0%	2	6%
Psychotic disorder due to cannabis	0	0%	1	3%	1	3%
***Comorbid psychiatric disorders***	***n***	***%***	***n***	***%***	***n***	***%***
Cannabis use disorder	7	23%	9	29%	16	52%
Alcohol use disorder	4	13%	0	0%	4	13%
Cocaine/Stimulant use disorder	1	3%	2	6%	3	10%
Hallucinogen use disorder	0	0%	1	3%	1	3%
ADHD / Personality disorder	2	6%	0	0%	2	6%
***Demographics***	***n***	***%***	***n***	***%***	***n***	***%***
Male	11	35%	11	35%	22	71%
Female	4	13%	5	16%	9	29%
Mean age at study initiation (years)	38.2 ± 11.9		32.19 ± 8.2		35.10 ± 10.6	
***Tobacco and cannabis use***						
CPD (self reported)	25.75 ± 8.3		19.46 ± 8.1		22.3 ± 8.3	
cannabis joints per day (self reported)	1.8 ± 1.7		2.06 ± 3.6		1.97 ± 3.1	

*ADHD, Attention deficit/hyperactivity syndrome; CPD, cigarettes per day*.

**Table 2:** Schematic representation of measurements on each assessment day (V1–V7).

	**V1**	**V2**	**V3**	**V4**	**V5**	**V6**	**V7**
***Measurements***	**day 0**	**day 7**	**day 14**	**day 21**	**day 28**	**day 91**	**day 175**
SCID	x						
PANSS	x	x	x	x	x	x	x
BDI	x	x	x	x	x	x	x
SWN-K	x	x	x	x	x	x	x
Brøset	x	x	x	x	x	x	x
Cigarette self-reports	x	x	x	x	x	x	x
Cannabis-joint self-reports	x	x	x	x	x	x	x
CBD-cigarette-count		x	x	x	x	x	x
Blood samples			x		x	x	x
Antipsychotic medication	x	x	x	x	x	x	x

*SCID, Structural Clinical Interview for DSM-IV; PANSS, Positive and Negative Syndrome Scale; BDI, Beck's Depression Inventory; SWN-K, Subjective Well-Being Under Neuroleptic Treatment Scale short form; Brøset, Brøset Violence Checklist*.

### Measures

At each assessment, the Positive and Negative Syndrome Scale (PANSS, scoring 1–7), the Brøset Violence Checklist, the Beck's Depression Inventory (BDI), and the Subjective Well-Being Under Neuroleptics Scale short form (SWN-K) were performed. The study physicians led the PANSS interviews, and nursing staff evaluated the Brøset Violence Scale on the acute wards. The participants filled out BDI and SWN-K questionnaires. We measured the quantity of the handed-out cigarettes (CBD and standard tobacco cigarettes) by the nursing staff, who entered each cigarette hand-out in respective participant forms. Additionally, we recorded self-reports concerning the quantity of tobacco, cannabis, and CBD consumption at each visitation. Blood samples measuring cannabidiol (CBD), cannabinol (CBN), tetrahydrocannabinol (THC), 11-hydroxytetrahydrocannabinol (THC-OH), and 11-nor-9-carboxy-tetrahydrocannabinol (THC-COOH) were taken at V3, V5, V6, and V7. The individual antipsychotic medication was recorded at each visitation (V1–V7). Moreover, the number of isolation events or enforced medication before and during study participation was registered.

### Blood Samples

Blood samples were prepared and analyzed by the forensic institute of Basel, Switzerland. Sampling procedures were essentially alike as previously published and described before (29). Solid-phase extraction employing the same protocol was either conducted manually or automatically using a Multi-Purpose Samples II (Gerstel GmbH, Mühlheim an der Ruhr, Germany). Analysis by gas chromatography coupled to tandem mass spectrometry was either conducted using a Trace GC Ultra related to a TSQ Quantum or a Trace 1,310 connected to a TSQ8000 (all instruments by Thermo Fisher Scientific, Waltham, USA).

### Antipsychotic Equivalents

For the conversion of the participants' antipsychotic medication, the Defined Daily Dose method by Leucht et al. (30) was applied (30). Each participants' antipsychotic medication was converted to olanzapine equivalents in mg per day using the antipsychotic dose conversion calculator provided by Leucht et al. (31).

### Primary and Secondary Outcomes

Primary outcomes were psychotic symptoms (PANSS), subjective well-being under neuroleptic medication (SWN-K), depressive symptoms (BDI), violent behavior (Brøset), and the amount of necessary antipsychotic drugs (olanzapine equivalents) with or without adjunctive CBD-cigarettes. Secondary outcomes were feasibility, treatment continuity, enforced medication, isolation events, tobacco, and cannabis use. Furthermore, the study aimed to correlate CBD and THC whole blood levels with psychotic symptoms via PANSS scores.

### Statistical Analysis

Descriptive statistical analysis for outcome parameters at day 0 (baseline), at 4 weeks (day 28), and at follow-up assessment II (day 175) were performed with IBM SPSS version 26. To analyze the temporal course of all primary outcomes, we used a discontinuous multilevel model (32), implying different linear trajectories for the active treatment and the follow-up phase, with a turning point set at the end of acute psychiatric treatment (on day 28). Note that multilevel models have been shown to provide more efficient and less biased results than complete case analyses or analyses in which missing values are imputed using the last observation carried forward method (33). Our model contained time (for active treatment and follow-up phase) and CBD-group (verum vs. placebo) as fixed effects, a random intercept parameter, and, if this improved model fit, random slope parameters for active treatment and follow-up phase. Differences between outcomes at specific time points (end of treatment and end of follow-up) were computed using contrast analyses. Outcomes were transformed, if necessary, to meet model assumptions. Thus, the Brøset was transformed using the function ln(x+1). Multilevel analysis was performed with “R” (34) using the nlme-package for mixed-effects models by Pinheiro et al. (35).

## Results

Of 31 included individuals, 16 were allocated to receive CBD-cigarettes, and 15 were randomized to the placebo (standard cigarette, non-CBD) group. Four participants withdrew from the CBD-group study during the interventional phase, and seven from the placebo group. Another participant from the placebo-group was referred to another medical facility for further somatic clarification. She presented pulmonary symptoms within the first few days of the study and was excluded from further participation. One participant from the CBD-group died unexpectedly from opiate intoxication (see 3.5. Adverse Events). After the acute therapy phase, two participants were lost to follow-up, and one participant withdrew from the CBD-group. Two were lost to follow-up from the non-CBD-group. The dropout rate in the CBD-group during the intervention 25% (including the death 31.25%), while the dropout rate in the placebo-group was 53.33%, without being significant (*p* = 0.11).

### Primary Outcomes

#### Descriptive Statistics and Multilevel Analysis

**Figure 2:**
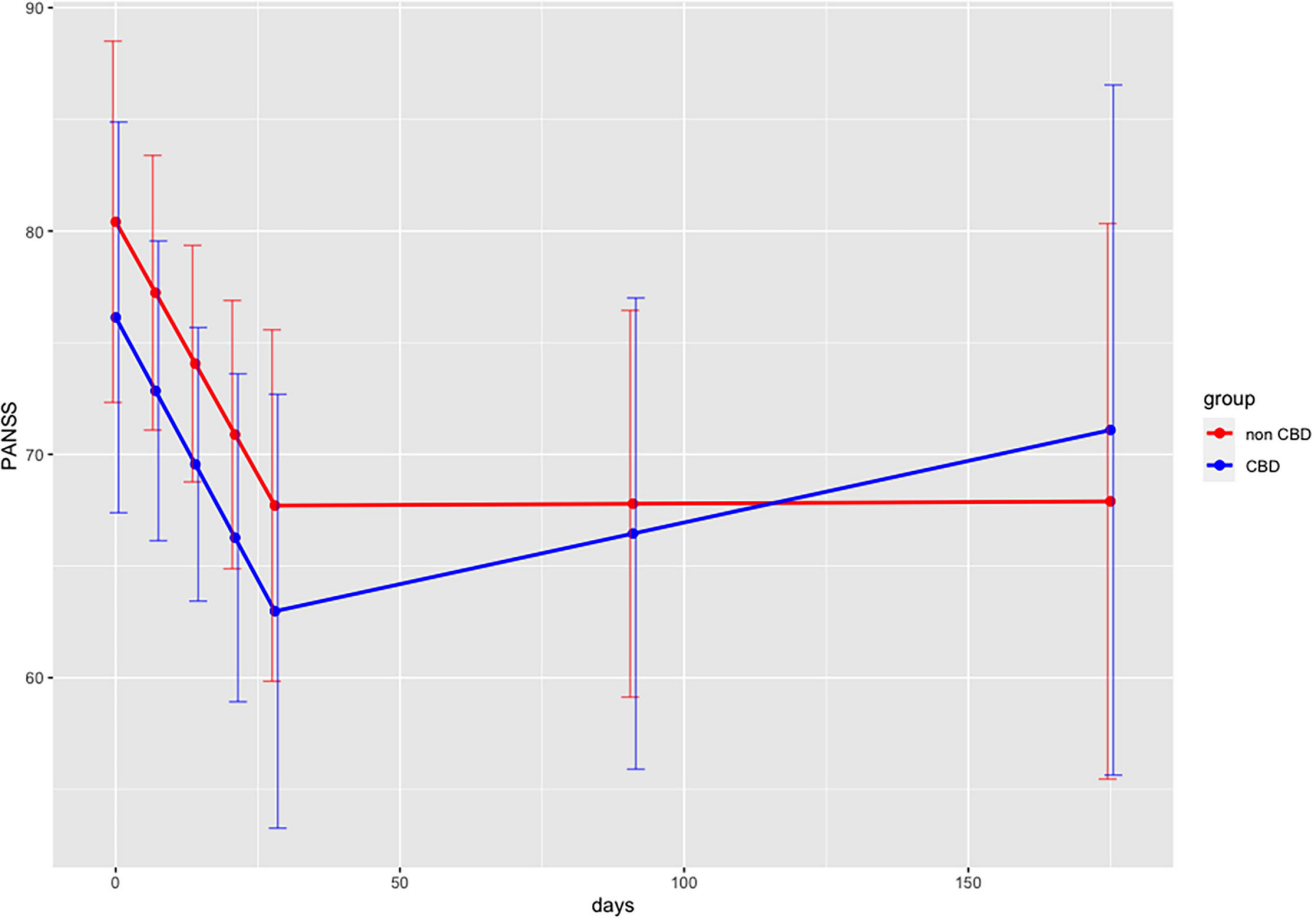
Predicted PANSS scores (in points) for interaction effects for both groups over time (days) with discontinuous multilevel model. Interaction days x group during acute therapy (days 0–28), Interaction days x group follow-up period (days 28–175); PANSS, Positive and Negative Syndrome Scale Scores; days, days 0–28 during acute therapy, days 28–175 follow-up period.

**Figure 3:**
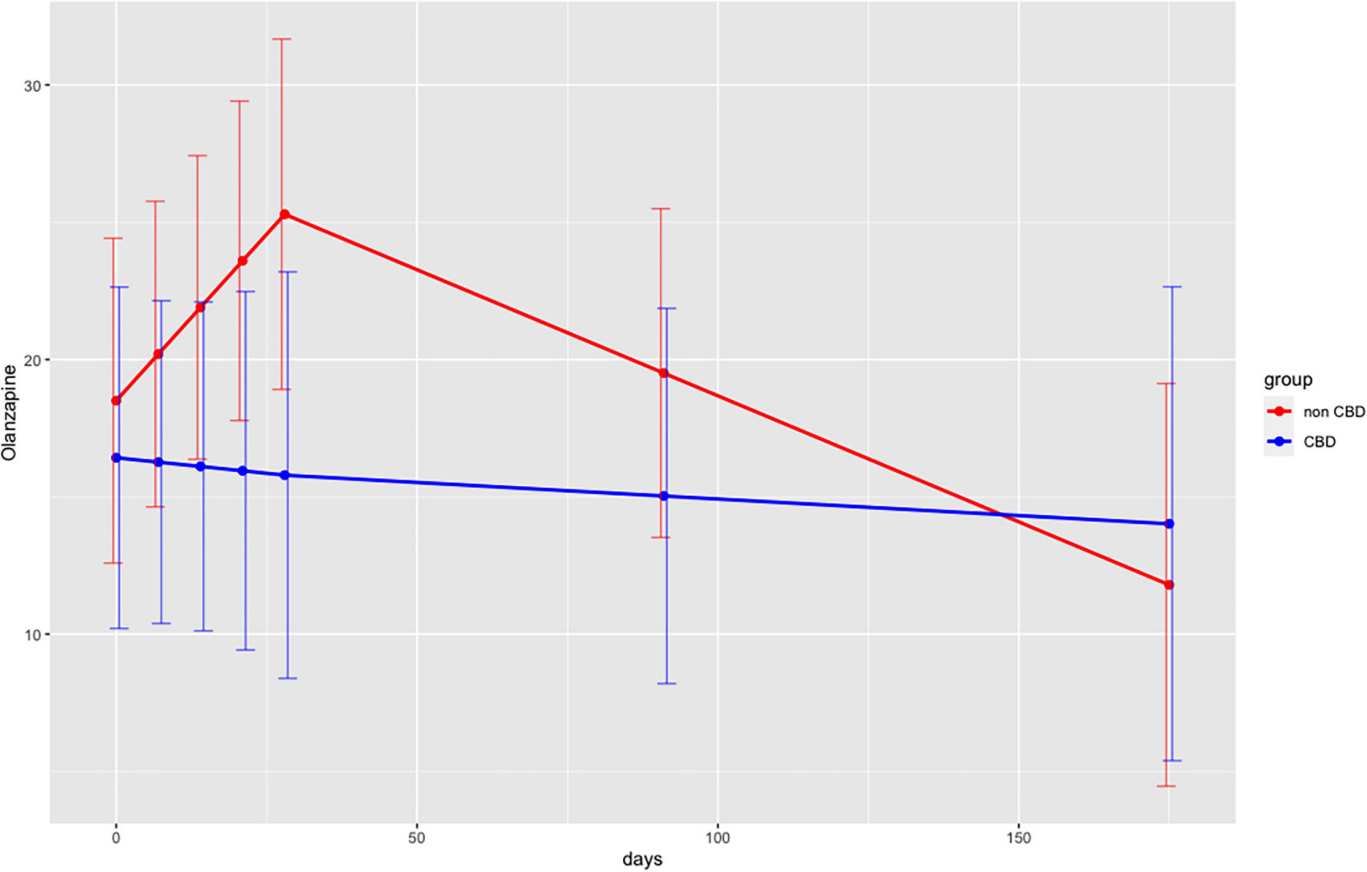
Predicted olanzapine equivalents (in mg) for both groups over time (days) with discontinuous multilevel model. Interaction days x group during acute therapy (days 0–28), Interaction days x group following acute therapy (days 28–175); PANSS, Positive and Negative Syndrome Scale Scores; days, days 0–28 during acute therapy, days 28–175 follow-up period.

Descriptive measures for all primary outcomes are displayed in Table 3 for days 0, end of acute therapy (day 28) and follow-up assessment II (day 175). Results from multilevel models are summarized in Table 4. For the outcomes PANSS, Brøset, and BDI, there was a linear decrease for both groups during the active treatment phase, but no linear trend during the subsequent follow-up phase (thus, the linear trend during follow-up was significantly more positive than that during the active treatment phase). More importantly, this pattern did not differ between the two groups. For the outcomes SWN-K and olanzapine equivalents, there were no temporal trends across the entire study period, and this pattern did not differ between the two groups. Patients in the verum group had consistently higher values than those in the placebo group (CBD-group main effect) for SWN-K, but not for olanzapine equivalents. Model-based temporal courses of PANSS and olanzapine equivalents are shown in Figures 2, 3. The high of drop-out rate and low number of remaining participants did not allow for further statistical analysis.

**Table 3:** Descriptive statistics for primary outcomes for day 0, 28, and 175.

		**Day 0**		**Day 28**		**Day 175**	
**Measures**	**Group**	***Mean***	***SD***	***Mean***	***SD***	***Mean***	***SD***
PANSS	CBD	79.00	11.82	65.73	15.85	69.00	23.46
PANSS	Non-CBD	79.64	21.94	69.17	16.96	62.00	11.60
BDI	CBD	13.29	6.96	5.91	5.68	13.63	8.18
BDI	Non-CBD	11.92	6.01	6.00	7.34	8.40	4.04
SWN-K	CBD	64.53	15.80	56.82	14.71	60.63	12.67
SWN-K	Non-CBD	50.75	13.29	48.00	23.76	49.40	13.09
Brøset	CBD	0.69	1.25	0.55	1.21	0.38	1.06
Brøset	Non-CBD	1.43	2.38	0.00	0.00	0.00	0.00
Olanzapine Eq	CBD	15.33	12.62	13.48	13.11	17.21	14.56
Olanzapine Eq	Non-CBD	18.69	12.62	28.46	6.14	12.09	5.34

*PANSS, Positive and Negative Syndrome Scale; BDI, Beck's Depression Inventory; SWN-K, Subjective Well-Being Under Neuroleptic Treatment Scale short form; Brøset, brøset violence checklist; Olanzapine Eq., Olanzapine equivalents; SD, Standard deviation*.

**Table 4:** Results from discontinuous model: interaction during (day 0–28) and following (day 28–175) acute therapy.

	**Interaction days x group** ***during*** **acute therapy**			**Interaction days x group** ***following*** **acute therapy**		
	**days 0–28**			**days 28–175**		
**Measure**	Coefficient^*^ (SD)	***t (df)***	***p*** **-value**	Coefficient^**^ (SD)	***t (df)***	***p*** **-value**
PANSS	0.016 (0.334)	0.048 (105)	0.962	−0.070 (0.348)	−0.201 (105)	0.841
BDI	0.122 (0.094)	1.297 (98)	0.198	−0.175 (0.107)	−1.633 (98)	0.106
SWN-K	0.156 (0.283)	0.533 (99)	0.595	−0.187 (0.328)	−0.570 (99)	0.570
Brøset	−0.010 (0.005)	−1.909 (105)	0.059	0.010 (0.006)	1.699 (105)	0.092
PANSS neg.	0.018 (0.087)	0.210 (105)	0.834	−0.041 (0.099)	−0.417 (105)	0.678
PANSS pos.	−0.002 (0.073)	−0.031 (105)	0.976	−0.008 (0.083)	−0.091 (105)	0.928
Olanz. Eq	0.265 (0.156)	1.704 (101)	0.091	−0.345 (0.177)	−1.944 (101)	0.054

* *Coefficients denote the differences in the slopes during therapy between the CBD and the non-CBD group*.

** *Coefficients denote the differences in the slope changes from therapy and follow-up period between the CBD and the non-CBD group. PANSS, Positive and Negative Syndrome Scale; BDI, Beck's Depression Inventory, SWN-K, Subjective Well-Being Under Neuroleptic Treatment Scale short form, Brøset, Brøset Violence Checklist, Olanz. Eq., Olanzapine equivalents, neg, negative; pos, positive*.

### Secondary Outcomes

#### Tobacco, Cannabis, and CBD Use

No significant differences for to cigarettes tal cigarettes per day (CPD) nor regular cannabis consumption were found. However, only the interventional products (CBD- and standard cigarettes) were recorded *via* staff. For daily tobacco and illicit cannabis use, we relied on self-reports. In the CBD-group, the average number of CBD-cigarettes per day was 9.70 (SD = 8.25) at day 28.

#### Enforcement Measures

During the acute therapy phase (day 0–28), we recorded no enforcement measures in the CBD-group, while nine isolations and one enforced medication event were registered in the non-CBD group.

#### Correlation of CBD and THC Whole Blood Levels With PANSS

Results from the multilevel model for CBD (μg/l) whole blood levels vs. PANSS revealed a slight negative, non-significant (*t* = −0.434, *p* = *0.6*67) correlation. For THC (μg/l) levels vs. PANSS a slight positive, non-significant correlation (*t* = 0.351, *p* = *0.7*28) was found in the multilevel analysis.

### Adverse Events

Generally, participants tolerated the CBD-cigarettes well. One individual claimed headaches (six, 25 %), discontinued CBD-cigarettes and withdrew from further study participation. The headaches were reported as mild and resolved after discontinuation of CBD-cigarettes. We recorded one serious adverse event (SAE). One participant in the CBD-group died. The patient was male and 41 years of age. He had a history of opioid and alcohol use. At the time of study participation, the patient received no pharmacological treatment for Opioid or Alcohol Use Disorder. Psychiatric diagnoses were paranoid schizophrenia, Tobacco Use Disorder, and Cannabis Use Disorder. Additionally, the patient had chronic obstructive pulmonary disease (COPD I) and psoriasis Vulgaris. We recorded no adverse events during the first 4 weeks of study participation. The day before his death, the patient was absent from the psychiatric ward during the day. Our nursing staff found the patient dead in his bed on their morning ward round on the following day. Prior blood analyses and ECG showed no pathological findings, explaining the SAE. Daily oral medication consisted of amisulpride 600 mg/d, olanzapine 30 mg/d, diazepam 10 mg/d, pantoprazole 20 mg/d, and B-Vitamins. This medication had been established and well-tolerated for 2 weeks. The patient had been smoking cannabis and tobacco for years daily. An autopsy conducted by the University Hospital of Basel's pathological institute found opiate intoxication as the cause of death. The autopsy revealed mildly pronounced coronary sclerosis, atherosclerosis, pulmonary sclerosis, and increased liver consistency. They also found moderately pronounced chronic bronchitis. The autopsy could not detect brain edema but signs of cerebral hypoxia. Toxicological forensic analysis revealed increased opiate concentrations. In synopsis, these results correlate with respiratory depression in the context of opiate intoxication. The pathological institute ruled out CBD intoxication as the cause of death.

## Discussion

### Differences to Previous Studies

Previous studies investigated the effects of orally administered CBD, either as a fixed single dose or fixed daily dose (12–16). Our study, in contrast, aimed at evaluating the antipsychotic efficacy of smoked CBD-cigarettes as an on-demand medication in addition to standard psychiatric care.

### Primary Outcomes

PANSS scores lowered both in the CBD-group and in the non-CBD-group. A similar pattern was found for BDI scores. Results from the discontinuous multilevel model revealed no significant group differences for all primary outcomes. The model showed consistently higher SWN-K scores and lower olanzapine equivalents for the CBD-group. These findings may point out to an antipsychotic and “neuroleptic medication sparing effect” of CBD-cigarettes. However, given the limited data available, it remains unclear whether these observations were due to chance or confounding factors. A biasing factor could be the physician's awareness of the patients receiving placebo and therefore increasing the antipsychotic medication. Furthermore, certain patient characteristics such as metabolization profiles may have influenced these observations. In the RCT conducted by Leweke et al. (15) found a similar reduction of PANSS scores for both groups comparing the effects of oral amisulpride (800 mg/d) vs. CBD (600 mg/d) in patients with acute schizophrenia. However, their study design did not allow for continuing illicit cannabis use among their patients, whereas our study aimed at evaluating the effects of smoked CBD within a naturalistic setting. Obviously, the consumption of illicit cannabis was not encouraged, but also not a restriction for study participation. Data suggests that simultaneous administration of CBD might potentiate the impact of THC (5). Hence, patient data within our study sample, who remained using cannabis high in THC during the trial, may have influenced our results. Nevertheless, studies simulating naturalistic settings, are needed, especially given the high prevalence comorbid cannabis use among patients with psychosis (36). Also, we did not exclude treatment-resistant patients, unlike studies conducted by Leweke et al. (15) and McGuire et al. (12), and the case report by Zuardi et al. (37), which may have confounded our results. The heterogeneity of different subpopulations, such as different substance use behaviors and stages of illness (19), as well as the open-label/on-demand CBD-medication may have influenced the outcomes of this study and should be more rigorously addressed in the future.

### Secondary Outcomes

The main problems of the study design were the impossibility to blind the study product and to predetermine the applied dose of CBD. Although the current study was designed to stimulate naturalistic conditions (investigation of an open-market product as adjunctive on-demand medication), a more rigorous study design with blinding procedures and fixed daily doses may have yielded different results and should be preferred for future studies. The treatment continuity was slightly higher in the CBD-group compared to the placebo group (see 4.5 Limitations). No enforcement measures were necessary for the CBD-group within the acute treatment phase, but nine enforcement events were recorded for the non-CBD-group. These observations might point out a reduced necessity of enforcement measures and increased compliance in the CBD-group.

### Adverse Events

Across the available clinical data, CBD implementation for psychiatric conditions generally causes low rates of AEs, the most common being diarrhea, nausea, tiredness, and hepatotoxicity (38). Especially drug-drug interactions must be carefully considered according to a recent systematic analysis by Huestis et al. (38). In the rhesus monkey LD50 for CBD intravenously was 212 mg/kg (39). Our study participants smoked on average 9.7 of our interventional CBD-cigarettes per day (at day 28). The individual who died from opiate intoxication smoked 20 CBD-cigarettes per day and had a bodyweight of 64.1 kg. 20 CBD-cigarettes contain ~ 400 mg of CBD, thus about 6.24 mg/kg, which is within the range of previous clinical studies (38). To our knowledge, no human fatalities associated with CBD have been reported. For example, McGuire et al. (12), found mild AEs for CBD 1000 mg/day in about a third of their patients, which was similar to the AE incidence in the placebo group (12).

### Limitations

This study had several limitations. The major limitation was the lack of blinding. As described above (2.2 Interventional product) blinding of the interventional product in this study was impossible, due to the intense odor of the CBD-cigarettes and the available placebo product (standard tobacco cigarettes). For future studies assessing the effects of smoked CBD blinding might be achieved by handing out cannabis cigarettes with a high content of CBD and THC <1% as *verum* and similar cannabis cigarettes with both CBD and THC <1% as adequate *placebo*.

A further significant limitation was the low number of included patients and a high dropout rate, impeding statistical power. Dropout rates in antipsychotic trials have varied between 19 and 74%, while second-generation antipsychotic studies and short trial periods have shown lower dropout rates (40). The higher number of dropouts in the non-CBD group may be explained by the disappointment of some individuals not being randomized in the CBD-group and therefore not seeing the benefit of participation. Also, the standard cigarettes handed out as placebo were not well-accepted by some participants. For future studies, obtaining the participants preferred cigarette type as placebo product might be beneficial. Also, our study population consisted of “moderately ill” patients (mean PANSS 79.30), whereas “markedly ill” corresponds to an average baseline PANSS of 96 or higher (28). This further limits the quality of the data and future designs should aim at generally applied PANSS thresholds for antipsychotic treatment trials.

Another limitation was reduced compliance for blood samples to quantify cannabinoid levels. Furthermore, no anandamide levels were measured, as did Leweke et al. (15). The use of other cannabis products (legal and illicit) in both the CBD-group and the non-CBD-group might have influenced results. Moreover, the concentration of CBD (approx. 20 mg per cigarette) might have resulted in an underdosing, compared to other studies (12, 15). The company “Heimat” only recently released pure CBD-cigarettes (approx. 60 mg of CBD per cigarette) after our trial had already started. Higher content of CBD per cigarette might have yielded different results.

## Conclusions

The main group effects in the discontinuous multilevel model were higher subjective well-being and less overall antipsychotic medication use throughout the acute therapy for the CBD-group. These results may suggest an antipsychotic medication sparing effect of CBD-cigarettes as adjunctive therapy in acutely psychotic patients. However, the open-label design, the impossibility of a fixed dosing regimen, and the low participation in the study affect the validity of the results. Smoked CBD might offer a harm-reductive intervention in psychotic patients with tobacco dependency and comorbid cannabis use. However, future studies with more rigorous study designs and larger samples are needed.

## Data Availability Statement

The raw data supporting the conclusions of this article will be made available by the authors, without undue reservation.

## Ethics Statement

The studies involving human participants were reviewed and approved by the Swiss Ethics Committee (Ethikkommission Nordwest- und Zentralschweiz, EKNZ). The patients/participants provided their written informed consent to participate in this study.

## Author Contributions

PK: drafted the manuscript. EL and SB: designed the study. EL: prepared the study. PK, EL, and V-NT: conducted participant assessments and collected the data. FD: supervised psychiatric treatment of participants and assisted in data collection. KM-C-B and PF: analyzed THC and CBD and metabolite levels. CH: provided substantial intellectual input. SB: led the study process and the writing of the manuscript. PK and EL: contributed equally to the realization and the preparation for the publication of this study. All authors contributed to the article and approved the submitted version.

## Funding

Heimat standard tobacco and CBD-cigarettes were funded by the Gertrud Thalmann Fonds of the Psychiatric University Clinics of Basel (UPK) for scientific assessment. The cigarette manufacturer Heimat and the Gertrud Thalmann Fonds had no further role in study design, data collection, or interpretation of results or publication process of this article.

## Conflict of Interest

The authors declare that the research was conducted in the absence of any commercial or financial relationships that could be construed as a potential conflict of interest.

## Publisher's Note

All claims expressed in this article are solely those of the authors and do not necessarily represent those of their affiliated organizations, or those of the publisher, the editors and the reviewers. Any product that may be evaluated in this article, or claim that may be made by its manufacturer, is not guaranteed or endorsed by the publisher.
